# An Unusual Side Effect of the COVID-19 Vaccine: A Possible Trigger of Polymyalgia Rheumatica

**DOI:** 10.7759/cureus.26617

**Published:** 2022-07-06

**Authors:** Anam Ahmad, Donica L Baker

**Affiliations:** 1 Internal Medicine, St. Luke's Hospital, Chesterfield, USA; 2 Rheumatology, St. Luke's Hospital, Chesterfield, USA

**Keywords:** myalgia, bnt162b2 vaccine, side effect of vaccine, covid-19 vaccine, pmr

## Abstract

Polymyalgia rheumatica (PMR) is characterized by myalgia and severe stiffness with decreased range of motion in the shoulder and pelvic girdles. The efficacy of the BNT 162b2 Pfizer vaccine has been proven and has been well-tolerated by patients; however, some instances of PMR have been reported after the COVID-19 vaccine. We are writing a case of a new-onset PMR in a 72-year-old woman with typical findings after receiving a booster dose of the BNT 162b2 Pfizer vaccine. This case report highlights that PMR should be in the differential diagnosis of myalgias caused by the vaccine.

## Introduction

Polymyalgia rheumatica (PMR) is an inflammatory condition typically affecting patients above 60 years old, characterized by myalgia and severe stiffness with decreased range of motion in the shoulder and pelvic girdles. Multiple genetic and environmental factors are involved in the pathogenesis of PMR and influence the susceptibility of the disease [1]. Certain viral and bacterial infections have been studied to have a temporal relationship with polymyalgia rheumatica and giant cell arteritis (GCA) by altering the immune system's balance [1]. Some instances of PMR after the influenza vaccine have been described previously [2,3]. Coronavirus disease 2019 (COVID-19) vaccine is essential to fight the lethal pandemic of severe acute respiratory syndrome coronavirus 2 (SARS-CoV-2) infection. Although the efficacy of the BioNTech-Pfizer COVID 19 vaccine (BNT 162b2) has been proven and has been well-tolerated by patients with chronic rheumatic diseases, some new-onset autoinflammatory conditions and disease flare-ups have been reported after administration of the vaccine [4,5]. 

## Case presentation

Our patient is a 72-year-old woman who was referred to the rheumatology clinic for widespread pain throughout her body and extremely elevated erythrocyte sedimentation rate (ESR) and C-reactive protein (CRP). Her past medical history was significant for autoimmune hepatitis, hypothyroidism, atrial fibrillation, hypertension, hyperlipidemia, cerebrovascular accident, stomach ulcers, irritable bowel syndrome, migraines, chronic urinary tract infection, depression, and anxiety. The patient mentioned that about 10 days after receiving the BioNTech-Pfizer COVID-19 booster vaccine (BNT 162b2), she started developing extreme fatigue, widespread pain throughout her body, and generalized weakness. She mentioned severe pain and stiffness in the shoulder and hip girdle to the point that she was unable to get up from the bed and had to use a cane for ambulation. She also endorsed a band-like headache around her temples as well as blurry vision. No jaw claudication symptoms were reported.

Initially, she had flu-like symptoms after the COVID-19 booster that gradually resolved, but the body aches and fatigue continued. Her musculoskeletal examination showed decreased range of motion in the shoulder and hip joints but was unremarkable for any joint effusion, synovitis, or tenderness. Muscle strength was 5/5 in the upper and lower extremities. No temporal tenderness or bulging was noted. Initial laboratory studies showed normal complete blood count (Table 1), basic metabolic profile, and thyroid function tests (Table 2). Her Inflammatory markers were extremely elevated; ESR was 108 millimeters/hour and CRP was 150 milligram/liter (Table 3). With high clinical suspicion of PMR and giant cell arteritis (GCA), the patient was started on prednisone 60 mg daily, and a temporal artery biopsy was scheduled. She had left and right temporal artery biopsies, which were negative for any histopathological abnormalities indicating no GCA. She was instructed to taper her prednisone dose gradually. At her two-week follow-up appointment, the patient showed a significant response to prednisone treatment, with improvement in her shoulder and hip girdle pain and stiffness. Her ESR improved to 54 and her CRP improved to 29 (Table 4). Her headache and blurry vision persisted despite a negative temporal artery biopsy, and she was referred to an ophthalmologist for further evaluation of blurry vision. 

**Table 1:** Complete blood count

Laboratory test	Value	Reference Range
Hemoglobin (Hb)	13 gram/deciliter (g/dl)	(12-16.0 g/dl)
White blood cells (WBC)	5.0x10^6^/microliter	(4.5-5.5 x 10^6^/microliter)
Platelets	299x 10^6^/microliter	(150-450 x 10^6^/microliter)

**Table 2:** Basic metabolic profile and TSH TSH: thyroid-stimulating hormone

Laboratory test	Value	Reference Range
Sodium	136 milliequivalents/liter (mEq/L)	1 35-145 mEq/L)
Potassium	4.0 (mEq/L)	3.5-5.0 mEq/L)
Bicarb	25 (mEq/L)	23-29 mEq/L)
Chloride	107 (mEq/L)	95-105 mEq/L)
Creatinine	0.9 milligram/deciliter ( mg/dl)	0.6-1.2 mg/dl
Blood urea nitrogen	15 mg/dl	5-20 mg/dl
Thyroid-stimulating hormone	3.5 mil international units/liter ( mIU/L)	(0.5-5.0) mIU/L

**Table 3:** Inflammatory markers at time of presentation

Laboratory test	Value	Value
C-reactive protein (CRP)	50 milligram/liter (mg/L)	(0-10 mg/L)
Erythrocyte sedimentation rate (ESR)	108 millimeter/hour (mm/hr)	(female <20 mm/hr)

**Table 4:** Improvement of inflammatory markers after treatment

Laboratory test	Value	Value
C-reactive protein (CRP)	29 (mg/L)	(0-10 mg/L)
Erythrocyte sedimentation rate (ESR)	54 (mm/hr)	(female <20 mm/hr)

## Discussion

PMR and GCA are inflammatory conditions that often occur together, typically affecting middle-aged people above the age of 60. They are more prevalent in specific populations, such as patients of Scandinavian descent, and their incidence increases between 70-80 years of age [1].

There are various hypotheses involved in the pathogenesis of PMR, including genetic and environmental factors playing a significant role in triggering the autoimmune process [1]. In genetically predisposed people, a close temporal relationship between parvovirus B19, Mycoplasma pneumonia, and Chlamydia pneumonia has been found [6]. Certain vaccines, like the Influenza A vaccine, have been previously associated with GCA and PMR in some cases [2,7]. Although the exact mechanism is unknown, Watad et al. found that vaccines activate the adaptive immune system leading to an autoinflammatory process [8]. Recently, there have been a few other case reports of PMR/GCA after COVID-19 vaccination as well. There is an ongoing study to understand the relationship between COVID-19 vaccination and the potential development of PMR/GCA [9]. Our patient also developed new-onset typical PMR symptoms almost 10 days after getting the booster shot of the vaccine.

Patients with PMR experience pain in the bilateral proximal muscle groups, which usually worsens with movement. Pain is also associated with prolonged morning joint stiffness and a restricted range of motion. Systemic symptoms like fever, malaise, fatigue, and anorexia can be present in around one-third of the patients. Patients with GCA have headaches more pronounced in temporal regions with or without the association of visual changes and jaw claudication [10].

The physical examination is usually significant for joint effusions, tenderness, and decreased passive range of motion. Muscle strength is usually normal. In the case of GCA, the temporal arteries may be thickened, nodular, tender, and occasionally with a bulging appearance [11].

The characteristic laboratory findings are elevated ESR, usually greater than 100, and elevated CRP. The CRP is a more sensitive indicator than ESR [12]. Patients showing systemic symptoms can have mild to moderate anaemia and slightly abnormal liver function tests. Markedly elevated ESR should raise the suspicion of GCA. 

Diagnostic criteria include age above 50 years, bilateral pain in proximal muscle groups, prolonged morning joint stiffness lasting more than 45 minutes for at least two weeks, elevated inflammatory markers (ESR or CRP); and prompt response to glucocorticoids [13]. Joint ultrasound (US) and resonant magnetic imaging (MRI) can help identify synovitis and peri-articular bursitis, and tendonitis associated with PMR but are not required for the diagnosis [14]. If GCA is suspected, temporal artery biopsy, temporal artery US, computed tomography (CT) scan, and positron emission tomography (PET) can be obtained. 

Management of PMR includes corticosteroids as the treatment of choice. For PMR, lower doses of prednisone 10-20 mg daily are adequate, while for GCA, higher doses of prednisone 40-60 mg daily are required. Pulse doses of intravenous methylprednisolone (1000 mg daily) may be given to patients with suspected impending visual loss [4]. Corticosteroids may prevent but cannot reverse visual loss. Generally, response to steroids is rapid, and symptoms improve within days. 

## Conclusions

The temporal relationship between COVID-19 vaccination and PMR has yet to be established, but our case report will add to the literature review. We hypothesize that the COVID-19 vaccine triggered the autoinflammatory state in our patient leading to PMR. Myalgias are a commonly reported adverse effect of the COVID-19 vaccine. We emphasize that physicians should pay attention to persistent myalgias and obtain ESR and CRP levels when appropriate to evaluate possible PMR, which response well to corticosteroid treatment. However, these cases are rare and should not prevent the population from receiving the vaccine, as the benefits of the COVID-19 vaccine outweigh the risks. 
